# Implementation and evaluation of a multi-professional care pathway and network for the promotion of needs-oriented, resident-oriented exercise therapy for oncological patients (MOVE-ONKO): protocol of a mixed-methods cohort study

**DOI:** 10.1186/s12885-025-13797-7

**Published:** 2025-03-13

**Authors:** Saskia Blütgen, Katja Krug, Katharina Graf, Ulrich Betz, Julian Böhm, Elke Jäger, Verena Krell, Jana Müller, Antonia Pahl, Annelie Voland, Sandra Weigmann-Faßbender, Anna Zinkevich, Michel Wensing, Lena Ansmann, Joachim Wiskemann

**Affiliations:** 1 Faculty of Medicine and University Hospital Cologne, Institute of Medical Sociology, Health Services Research and Rehabilitation Science (IMVR), Chair of Medical Sociology, University of Cologne, Cologne, Germany; 2 Department of Primary Care and Health Services Research, University Hospital Heidelberg, Im Neuenheimer Feld 130.3, Heidelberg, 69120 Germany; 3 Department for Oncology and Hematology, Working Group Exercise Oncology, University Cancer Center (UCT) Frankfurt, Krankenhaus Nordwest, Steinbacher Hohl 2-26, Frankfurt am Main, 60488 Germany; 4 Institute of Physical Therapy, Prevention and Rehabilitation, University Medical Center of the Johannes Gutenberg University Mainz, Mainz, Germany; 5 Department of Sports Medicine, University Medicine Tübingen, Hoppe-Seyler-Str. 6, Tübingen, 72076 Germany; 6 Department for Oncology and Hematology, University Cancer Center (UCT) Frankfurt, Krankenhaus Nordwest, Steinbacher Hohl 2-26, Frankfurt am Main, 60488 Germany; 7 Department of Sports Medicine, Charité – Universitätsmedizin Berlin, Philippstr. 13, Berlin, 10115 Germany; 8 Department of Medical Oncology, University Hospital Heidelberg, National Center for Tumor Diseases (NCT) Heidelberg, Im Neuenheimer Feld 460, Heidelberg, 69120 Germany; 9 Department of Medicine I, Hematology, Oncology, and Stem-Cell Transplantation, Medical Center, Faculty of Medicine, University of Freiburg, Freiburg, Germany; 10 National Center for Tumor Diseases Dresden (NCT/UCC), Dresden, Germany

**Keywords:** Cancer, Care pathway, Exercise therapy, Evaluation, Implementation

## Abstract

**Background:**

Physical activity has been shown to be beneficial for people diagnosed with cancer. MOVE-ONKO aims at implementing a new multiprofessional care pathway in 3 large regions in Germany at the interface of oncological treatment and exercise therapy for cancer patients. The related study aims to evaluate the implementation processes and patient outcomes.

**Methods:**

The project comprises 3 phases: 1) preparation, 2) implementation in Comprehensive Cancer Centres (CCCs), 3) implementation in remote cancer centres. In phase 1, individual prerequisites for the implementation are assessed in the participating CCCs. The care pathway which is supported by digital/app-based exercise oncology health record is developed and adapted to the CCCs. Phases 2 and 3 focus on the implementation and evaluation of the care pathway.

To study the effectiveness, primarily changes in patients' health-related quality of life are examined in a prospective design. Secondary outcomes include physical activity behaviour, motivation for physical activity and patient activation. A sample of 2240 cancer patients ≥ 18 years (*n* = 280 patients at seven CCCs in phase 2 and *n* = 1.960 patients at the seven CCCs and 49 remote oncological centres in phase 3) are recruited for assessment at inclusion and at 4, 12 and 24 weeks.

The newly implemented care structures and processes will be assessed from the perspective of key informants, healthcare providers and patients. The perspectives of key informants on the implementation from the CCCs will be recorded in a prospective design based on a questionnaire and supplemented by in-depth semi-structured interviews across the three phases. The implementation processes are documented by assessing existing structures in CCCs. In phase 2 (*n* = 105) and phase 3 (*n* = 350), healthcare providers will be surveyed with questionnaires. Based on purposeful sampling (*n* = 18) will be interviewed. Additionally, qualitative patient interviews will be conducted about their experiences in the care pathway.

**Discussion:**

A successful implementation of the care pathway as part of the study will contribute to the growing evidence on the implementation of a nationwide structured approach to exercise therapy for patients with cancer.

**Trial registration:**

ClinicalTrials.gov (ClinicalTrials Identifier NCT06185777) at December 29, 2023.

## Background

Interventions to enhance regular physical activity, especially as systematic and needs-based exercise therapy, have proven to be an effective supportive care element for people diagnosed with cancer in all stages of the disease, as shown in more than 700 randomized controlled trials with about 50.000 patients [1, 2]. International guidelines and consensus recommendations [1, 3, 4] call for multiprofessional approaches to promote physical activity in cancer patients and propose ways to plan and carry out needs-based exercise therapy. In Germany, exercise therapy for cancer patients is recommended in S3 guidelines for supportive therapy for prostate and breast cancer [5–7]. Despite the overwhelming evidence, concepts and implementation strategies to integrate exercise therapy and physical activity counselling into oncological care remain sparse [1–3, 8–11].

Several barriers for implementing exercise therapy have been identified: foremost, integrating exercise therapy in acute oncological care and access to exercise therapy programmes due to lack of funding and counselling resources. The integration of exercise therapy comprises early information and physical activity counselling. General recommendations for exercise are challenged by the individual needs and capabilities of patients, which are related to symptoms of the disease, side effects of the oncological treatment and individual preferences [1, 4, 12]. Therefore, healthcare providers need to consider the individual situation of the cancer patient in physical activity counselling. Many healthcare providers lack competencies to inform patients about exercise therapy. Additionally, time is limited in the busy clinical schedule [10, 13]. Healthcare providers often underestimate patients’ resources and capabilities and are unaware of available information and experts for exercise therapy within their healthcare organization [11, 14, 15]. Activity-focused conversations are postponed or omitted forfeiting shared decision-making for supportive exercise therapy. Thus, the adoption of physically-active behaviour by cancer patients is limited [14, 16, 17].

Additionally, adequate exercise therapy programmes of high quality are often not available or easily accessible for all patients. These barriers were also assessed in a recent study in Germany evaluating a network providing exercise therapy [18]. Other barriers identified were complexity of certification for exercise therapy in oncology and missing network structures, travel time for patients, missing referrals, and lack of knowledge of healthcare staff as well as missing certification and lack of knowledge of exercise therapists without specialisation in cancer.

Therefore, the aim of the MOVE-ONKO project is to implement and evaluate a healthcare delivery model to connect cancer patients with exercise therapy which is based on their individual needs and abilities. A structured approach—the MOVE-ONKO care pathway—identifies eligible cancer patients and provides information and guidance on evidence-based individual physical activity.

## Description of the MOVE-ONKO care pathway

**Fig. 1:**
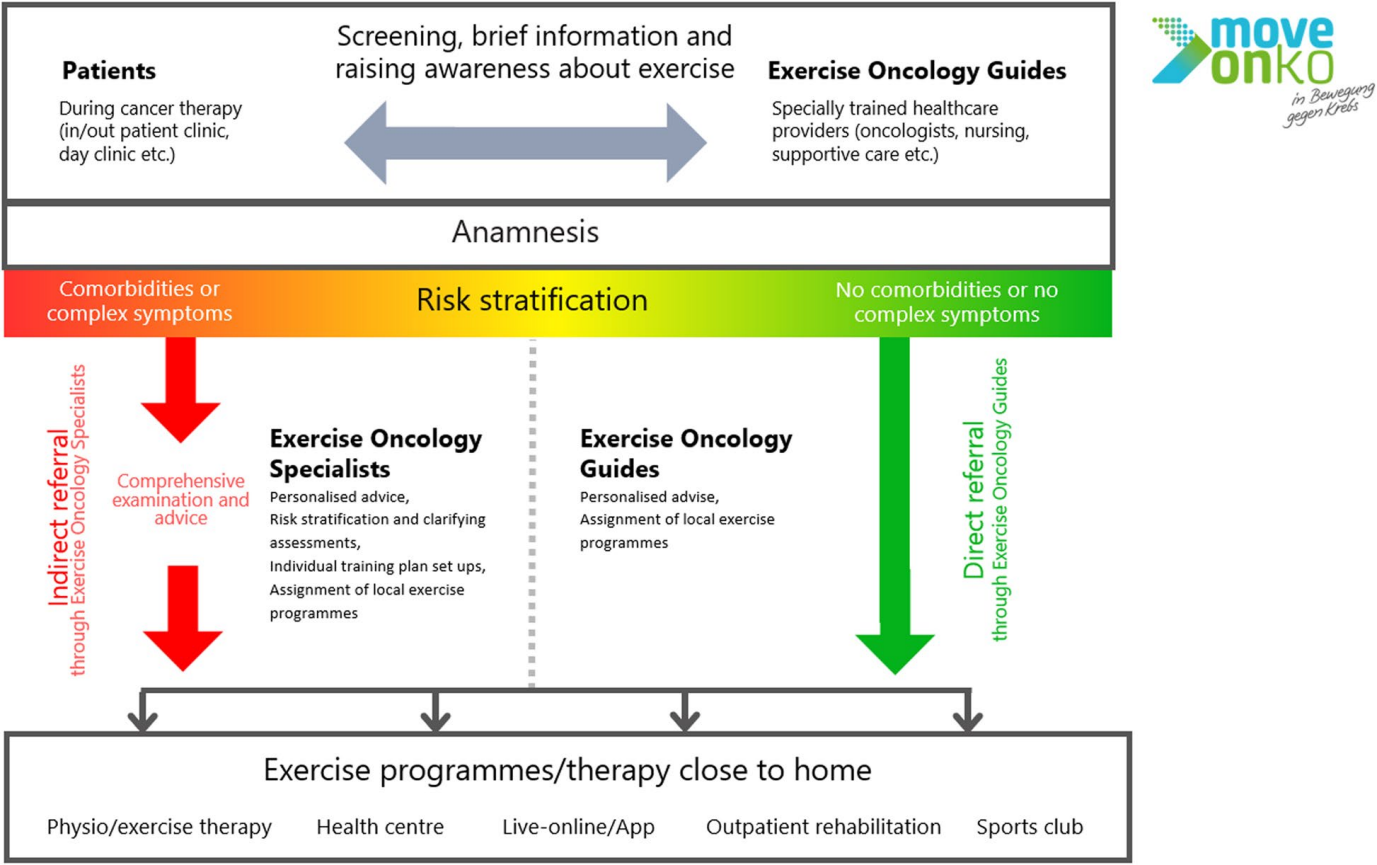
MOVE-ONKO care pathway

The care pathway (Fig. 1) describes a desired series of steps that patients follow during healthcare delivery, covering initial screening, followed by a brief counselling session to gain patients’ awareness and an intensive physical activity counselling. The pathway becomes part of healthcare delivery and is the intervention that is implemented in this project. At the same time, it is a strategy to implement evidence-based physical activity interventions in cancer care.

Patients are included into the pathway at the start or during the delivery of cancer treatment (medication, radiation, surgery, or combinations of these). They are approached by exercise oncology guides (EOGs). EOGs are oncology professionals with direct patient contact (e.g. in day clinics, oncology outpatient clinics or consultations). Patients can also be referred by physicians or other specialists; patients may also approach EOGs independently. The need for exercise therapy is determined on the basis of screening questions regarding fatigue, psychological stress, polyneuropathy, physical functioning, quality of life as well as on the basis of patients’ interest. If a patient is eligible, a detailed exercise-related history is taken, followed by a brief counselling session. The next step is by a risk assessment (modified risk assessment according to the National Comprehensive Cancer Network (NCCN) criteria and the American College of Sport Medicine (ACSM) pre-participation screening [19]) based on the clinical data collected.

If no risk factors, such as serious comorbidities or complex symptoms that are critical in relation to exercise therapy, are present, an EOG advises the patient on how to proceed. If risk factors or a more comprehensive counselling request are present, further counselling is provided by an exercise oncology specialist (EOS). The EOSs are exercise therapy professionals (exercise physiologist/scientists or sports physicians), who receive training in oncological content developed as part of the MOVE-ONKO project prior to the start of patient recruitment.

Based on individualised recommendations made during the physical activity counselling, patients are recommended to attend a specific exercise therapy option, such as structured exercise therapy programmes or community-based physical activity services, and virtual exercise therapy programmes by using the MOVE-ONKO APP. EOGs and EOSs refer patients to exercise therapy programmes by contacting local exercise therapy or physical activity options.

The care pathway is supported by the digital/app-based exercise oncology health record (MOVE-ONKO APP) which is used by EOGs and EOSs to guide them through the care pathway and to collect exercise therapy relevant clinical data. Thus, the MOVE-ONKO APP provides a common platform for patient files. The APP can also be used by patients to communicate with therapists and vice versa, especially when patients use the MOVE-ONKO APP-based exercise therapy programme. Additionally, a web-based information platform provides general information about cancer and physical activity as well as integrates an existing search platform to local exercise therapy services. There will be further support and feedback between these centres and researchers to support the implementation of MOVE-ONKO.

## Aims and research questions

The evaluation of the MOVE-ONKO care pathway focusses on the effectiveness of the intervention (intervention aim) and the uptake of the pathway by professionals and patients (implementation aim). Intervention success will be observed in benefits of physical activity and exercise therapy for the patients (e.g. better physical functioning). Implementation success as well as structures and processes for a long-term and sustainable implementation will be explored from the perspectives of patients, key informants and healthcare providers.

The evaluation research focusses on the following research questions:

Evaluation of intervention outcomes.

- How do patient-reported experiences and outcomes develop in patients enrolled in the MOVE-ONKO care pathway?

Evaluation of implementation processes.

1. To what extent can the planned MOVE-ONKO intervention be implemented in phases 2 and 3 in the CCCs and remote cancer centres?
2. Which barriers and facilitators determine the implementation in phases 2 and 3 of the MOVE-ONKO intervention?
3. Which resources guarantee/predict sustainable implementation in phase 2 and phase 3 of the MOVE-ONKO intervention?
4. To what extent does the MOVE-ONKO intervention increase the reach of the care pathway and the utilisation of exercise therapy by patients?

## Methods

### Logic model

**Fig. 2:**
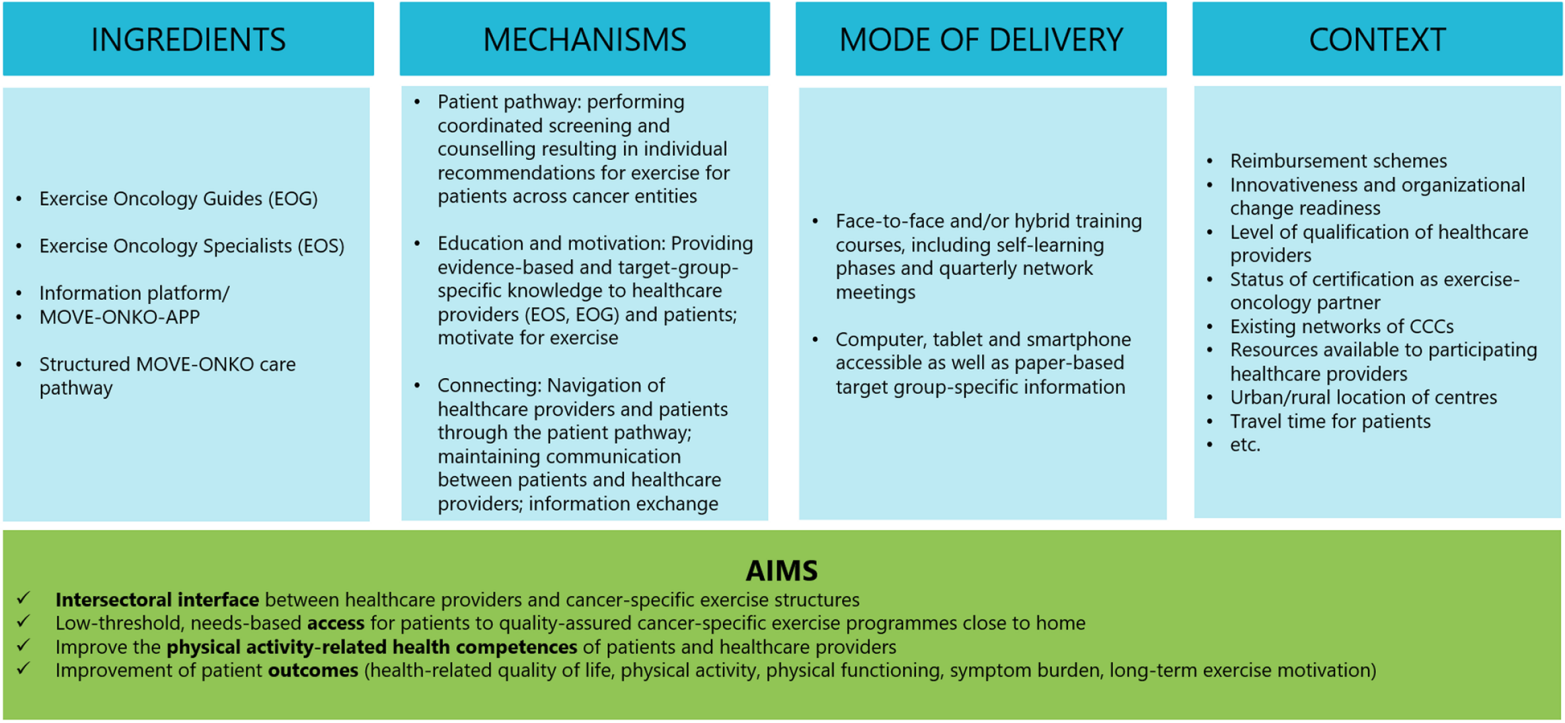
Logic model based on AIMD

The AIMD framework [20] was used to guide the evaluation. According to the framework aims, components, mechanisms of the intervention and delivery mode of the intervention are summarised in a logic model (Fig. 2). In order to consider the specific circumstances of the different participating centres, we have added a contextual component. The logic model of the MOVE-ONKO project was created in an iterative process involving programme developers and evaluators.

### Study design

The prospective observational mixed-methods study (without control arm) will combine perspectives of key informants, healthcare providers and patients based on survey data and in-depth semi-structured interviews.

**Fig. 3:**
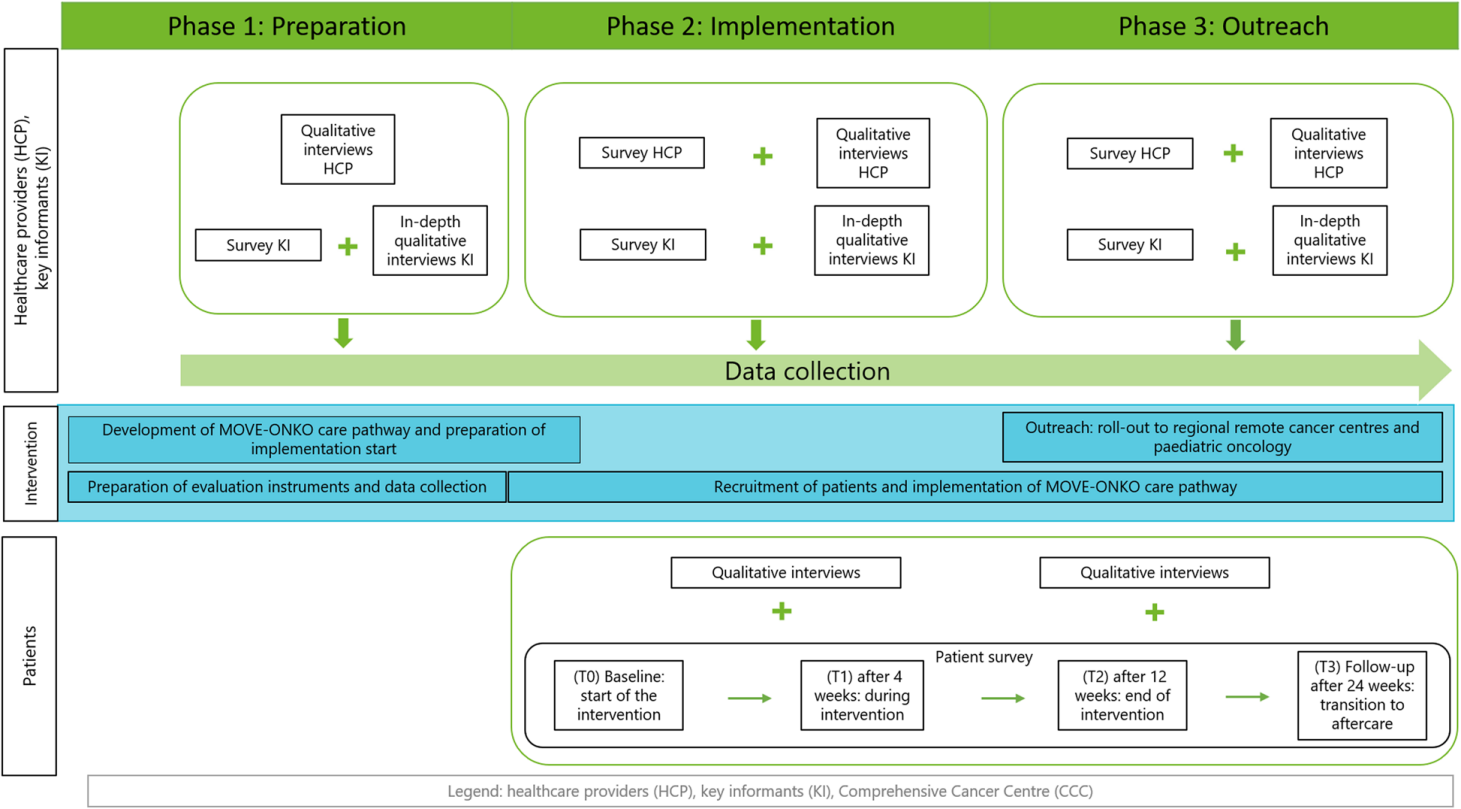
Evaluation design

The study comprises three phases (Fig. 3): 1) preparation, 2) implementation in seven Comprehensive Cancer Centres (CCCs) in three large regions in Germany, 3) implementation in remote cancer centres.

The care pathway will be developed in phase 1 and adapted to the CCCs. Additionally, specific prerequisites of the participating CCCs will be assessed. Phases 2 and 3 focus on the implementation of the care pathway. In phase 2, the care pathway will be implemented in seven CCCs; phase 3 will additionally include remote oncological centres associated with the regional CCCs. These can be organ-specific cancer centres or ambulatory oncological care organisations. The evaluation in both phases focuses on intervention outcomes and implementation processes. Intervention outcomes relate to patients’ reports on quality of life, physical activity and other outcomes. Implementation processes are examined from the perspectives of key informants, healthcare providers and patients.

## Evaluation of intervention outcomes

To observe intervention outcomes, a longitudinal prospective study without control group evaluates patient outcomes before, during and after the implementation of the care pathway. Patients receive questionnaires either electronically or paper-based at inclusion of the study before receiving physical activity counselling (t0), 4 weeks (t1), 12 weeks (t2) and 24 weeks (t3) after inclusion (Fig. 3).

### Participants and recruitment

Eligible for participation are cancer patients, at least 18 years old, with all cancer types regardless of the stage of the disease and during acute treatment. They show symptoms related to the disease or the treatment which have been shown to be positively impacted by exercise therapy (i.e. fatigue, emotional strain, polyneuropathy, reduced physical functioning, reduced quality of life) and/or they are interested in exercise therapy. Patients are included at all stages of their cancer treatment.

Patients are recruited for the study at the start or during the delivery of cancer treatment (medication, radiation, surgery, or combinations of these). They are approached by EOGs or referred by physicians or other specialists, patients may also approach EOGs or EOSs independently. Patients are informed about the study and the MOVE-ONKO care pathway. If they consent to participate in the study, they are asked to fill in the first survey and are enrolled in the MOVE-ONKO care pathway. Participation in the MOVE-ONKO care pathway involves participating in the study. There are no further incentives for the patients.

### Measures

Intervention outcomes are addressed in patient questionnaires comprising the European Organisation for Research and Treatment of Cancer Quality of Life Questionnaire (EORTC QLQ-C30 [21], primary outcome: subscale Physical functioning), the Godin-Shepard Leisure-Time Physical Activity Questionnaire [22], and a questionnaire to assess Physical Activity-Related Health Competence (PAHCO [23]) (Table 1). Additionally, sociodemographic characteristics, motivation for physical activity, social support, patient activation, experiences with the care pathway, patient involvement and shared decision making, and adherence to exercise therapy are assessed. Patient questionnaires will be pretested for practicability to avoid overstretching of participating cancer patients’ time and energy.

**Table 1:** Patient survey measures and data collection times

Outcome	Measure	Data collection time			
		Baseline (t0)	4-week follow-up (t1)	12-week follow-up (t2)	24-week follow-up (t3)
Physical functioning and quality of life	EORTC QLQ-C30 [21]	x		x	
	EORTC QLQ item bank items	x		x	x
Physical activity	Godin-Shephard Leisure Time Physical Activity Questionnaire [22]	x	x	x	x
Physical activity-related health competence	PAHCO questionnaire [23]	x	x	x	x
Motivation for physical activity (behaviour change)	HAPA Stages assessment: stage, duration of behaviour pattern performance	x	x	x	x
	HAPA Stages assessment: outcome expectancy	x		x	
	HAPA Stages assessment: intention	x		x	x
	HAPA Stages assessment: plans		x	x	x
Self-concordance of sport- and physical activity-related goals	SSK [24]	x	x	x	x
Social support	BS6 [25]	x			
Patient activation	PAM [26]	x		x	
Care assessment	PACIC (adapted) [27, 28]	x		x	
Experiences with MOVE-ONKO care pathway	PEACS (adapted) [29]		x		
Patient involvement and shared decision making	SDM-Q-9 (adapted) [30]		x		

*BS6* Brief Social Support Scale, *EORTC QLQ-C30* European Organisation for Research and Treatment of Cancer Quality of Life Questionnaire Core 30, *PAHCO* Physical Activity-related Health Competence, *HAPA* Health Action Process Approach, *PACIC* Patient Assessment of Chronic Illness Care, *PAM* Patient Activation Measure, *PEACS* Patients’ Experiences Across Health Care Sectors, *SDM-Q-9* Shared Decision-Making Questionnaire; t0 Baseline, t1 4-week follow-up, t2 12-week follow-up, t3 24-week follow-up

### Sample size calculation

The intervention is anticipated to cause a change in the subscale Physical functioning of the EORTC QLQ-C30 during the three-month intervention period between t0 and t2, with an effect size (ES) = 0.3 in repeated measures design. In phase 2, participants will be recruited across seven CCCs, and analyses will be performed across all centres. In phase 3, there will be 49 peripheral centres in addition to the seven CCCs. This brings up the total number of recruitment centres to 56 that will involve patients in the MOVE-ONKO care pathway and its evaluation. The following assumptions are considered: power = 0.80, intra-subject correlation = 0.5, intraclass correlation (ICC) = 0.01 (patients in centres), and drop-out = 10%. To demonstrate an effect of 0.3 between t0 and t2, a total of approximately 280 patients are required from seven centres (*n* = 40 per centre) in phase 2. In phase 3, around 1,960 patients are needed from 56 centres.

## Evaluation of implementation processes

Implementation processes (process evaluation) will be explored by assessing barriers and facilitators of a successful implementation, according to the RE-AIM Modell [31], Consolidated Framework for Implementation Science (CFIR) [32] and sustainability frameworks [33].

The purpose is to gain an overview of current practices (status quo) in the centres regarding physical activity interventions in cancer care, and to collect factors associated with these practices as well as the organisational context that may influence the quality of the implementation process. The evaluation additionally focusses on developing and extending the care pathway in the participating centres (CCCs and remote cancer centres).

The assessment of the extent of the implementation (adoption), sustainable implementation (maintenance) and reach follow the RE-AIM framework [31]. Maintenance as the implementation success is based on sustainability of implementation into routine care and its structures and processes in the CCCs. In addition, quality assurance will be evaluated on the basis of quality dimensions (structure, process and outcome quality). Quality assurance includes the use of the care pathway by patients (reach) and utilization.

Barriers, facilitators and suggestions for sustainability will be assessed in a longitudinal questionnaire and interview study conducted with healthcare providers and key informants at seven CCCs in three large regions in Germany (phases 1 and 2) and remote cancer centres (phase 3).

### Participants and recruitment

Key informants and healthcare providers will be surveyed in phases 2 and 3 to record potential changes during the implementation process and outreach. Key informants are defined as individuals with the comprehensive knowledge within their organisation. Healthcare providers include doctors and nurses, nutritionists, psycho-oncologists and social services.

As key informants, the leaders of the physical activity unit within their respective organisations (*n* = 7) are recruited. They will be invited to fill out a postal survey, followed by in-depth semi-structured interviews. The survey will be followed up with two reminders and in-depth semi-structured interviews will be conducted using the survey responses as a basis for the interviews. Key informants will be invited in all three phases to fill out a postal questionnaire followed by an interview. Phases 1 and 2 concentrate on CCCs key informants. The outreach in phase 3 into remote cancer centres will include further key informants. Potential remote centres are ambulatory care oncologists who will receive an incentive for participation.

In addition, each key informant will be requested to enlist at least three healthcare providers who work in the physical activity context for further surveys and interviews. Healthcare providers fulfilling the following criteria are eligible to participate:

- are at least 18 years old,
- have been in their current position for at least six months,
- work directly with patients, and
- possess a direct understanding of relevant care procedures, such as existing exercise therapy programmes and care pathways.

To ensure a comprehensive evaluation of all professionals involved into the implementation process, additional interviews with healthcare providers will be conducted in phase 1. In phases 2 and 3 survey and interviews will be conducted. The interviews will take place face-to-face at the CCCs or online according to preferences of the interviewees.

All patients enrolled in the MOVE-ONKO care pathway in phase 2 who filled in both baseline (t0) and 4-week follow-up (t1) questionnaires are eligible for participating in a patient interview. A purposeful sample will receive an invitation to an interview within 2 weeks of returning the t1 questionnaire. Interviews will take place online, via telephone or face-to-face at a place designated by the patient according to preferences of the interviewees.

### Measures

Quantitative survey and documentation: A questionnaire is created to survey key informants and healthcare providers based on the CFIR [34] and sustainability frameworks [33]. It includes both established, validated scales and self-developed items. Outcomes and measures are overlapping for both groups and are therefore presented together (Table 2). The questionnaire's development is part of the evaluation process and will be adapted to further phases as an iterative process. The survey will focus on indicators of structural quality, including the structural characteristics of the organisation and its respective units such as size and sponsorship, the exercise therapy unit's resources including staffing and qualifications. To assess process quality, data and information on organisational processes such as standardisation of patient care processes, existing exercise therapy services and existing collaborations and the current care pathway in the CCC will be collected. Moreover, indicators of organisational culture such as social capital [35], organizational change attitudes [36], and organisational health literacy [37] will be measured (Table 2). Socio-demographic information includes sex, age, profession, position within the organisation, job tenure, and professional experience in the relevant field will be collected for healthcare providers and key informants.

**Table 2:** Quantitative survey for healthcare providers and key informants

Outcomes	Measures	Data collection time	Participants
Structural characteristics	Self-developed questions on size and sponsorship	Phase 1/2/3	KI
Personal resources	Self-developed questions on qualifications	Phase 1/2/3	KI
Organisational culture	- SOCAPO-E [35] - Change Attitude Scale [36]	Phase 1/2/3	KI
Organisational health literacy	HLHO-10 [37]	Phase 1/2/3	KI
Equipment and Facilities	Self-developed questions on technical and spatial equipment	Phase 1/2/3	KI and HCP
Cooperation and networks	Self-developed questions	Phase 1/2/3	KI and HCP
Work intensity	Self-developed questions	Phase 1/2/3	KI and HCP
Physical activity-related health competences and knowledge and attitudes towards physical activity	PAHCO [23]	Phase 2/3	HCP
Technology commitment	Technology commitment (TB) [38]	Phase 2/3	KI and HCP
Sustainability	NoMAD [39, 40]	Phase 2/3	KI and HCP
Experience with MOVE-ONKO training and App use	Self-developed questions	Phase 2/3	HCP
Adherence	Self-developed questions on perceived motivation of the patients by healthcare providers and utilisation behaviour of the patients	Phase 2/3	HCP

*KI* key informants, *HCP* healthcare providers, *SOCAPO-E* Social capital of healthcare organizations reported by employees, *HLHO-10* health literate health care organization 10 item questionnaire, *PAHCO* physical activity-related health competence, *NoMAD* Normalisation Measure Development questionnaire, *TB* short scale for measuring technology commitment

Adoption [31] will focus on trained healthcare providers and patients. For healthcare providers, the number of trained EOGs and EOSs will be documented. During the study, the number of counselling encounters per trained person will be assessed. Feedback on the training in phase 2 (implementation in CCCs) will be used to give recommendations for implementation in phase 3 (outreach). It will also be monitored how the information platform and APP are used by healthcare providers and patients. APP and platform provide routine data. Sources for routine data in the APP are filled by questionnaires and anamnesis, as well as usage data, usage behaviour, and intervention documentation (therapy goals, training). The following criteria will be observed:

- number of screenings in CCCs and remote cancer centres
- number of included patients with complete anamneses before recommending an individual exercise therapy based on a shared decision as documented in the patient file
- number of included patients with an individual exercise therapy recommendation as documented in the patient file and reported by patients in questionnaire
- number of included patients taking up exercise therapy as documented in the patient file or reported by patients in questionnaire
- distance between residence and place of therapy for patients taking up exercise therapy as reported by patients in questionnaire
- number of patients who train via app or in the recruiting centres and receive support according to the therapy plan as documented in the APP and reported by patients in the questionnaire
- number of healthcare providers who interact via APP

Reach [31] comprises the availability of the care pathway for patients and the knowledge gain for participating healthcare providers. Availability will be described by assessing gross and net number of eligible patients and the number of actual patients included. The gross number are all eligible patients who are potential users of the care pathway, i.e. all cancer patients in the participating centres under acute treatment who are at least 18 years old. This number can be estimated from the yearly structured quality reports of each centre. The net number are all patients who can potentially be included given the resource constraints in the centres. There is a limited number of EOGs at the centres recruiting patients for the MOVE-ONKO care pathway. The maximum number of potential patients to be included by the MOVE-ONKO financed staff is derived from combining staff hours and estimated time for recruitment and physical activity counselling. Recruiting staff will document the number of patients approached for recruitment. Gross and net numbers will be compared with the number of actual recruitments taking patients’ willingness to participate into account. In the survey and interviews of key informants and healthcare providers knowledge gain will be measured by the spread of knowledge about exercise therapy in oncology supportive care in CCCs and remote cancer centres.

Qualitative interviews: Two separated interview guidelines are developed for key informants and healthcare provides based on theme complexes of a previous study [18] and on the CFIR framework [32]. Interview guides and questionnaires for key informants and healthcare providers will be further developed during the data collection process and results of previous phases. Interviews will take place at CCCs, remote centres or online. Both questionnaires and interview guides are pretested for comprehension and applicability.

The patient perspective of the implementation process will be evaluated with patient interviews. They will focus on experiences with the components of the care pathway: how they were addressed and informed, their impression of the physical activity counselling and of the information they were given, which recommendations they received, if they followed them and how they experienced the therapy. Additionally, patients will be asked to report on transfers within the pathway, i.e. EOG to EOS, counselling and recommendation to exercise therapy and physical activity, and on difficulties that arose including digital challenges when using the information platform or the MOVE-ONKO APP. Interview guidelines are based on research questions, discussed in the research team and adapted as necessary.

### Sample size calculation

Since no effects are analysed for the implementation outcomes, no sample size calculation is was made. The case number planning of the key informants and healthcare providers is determined by the project structure. The sampling of the key informants and healthcare providers is limited by the number of the seven cooperating CCCs and the associated 49 remote centres. All key informants will be included into the quantitative survey (phase 1/2: *n* = 7, phase 3: *n* = 56). Interviews will be conducted with CCCs key informants (*n* = 7) in all phases and in phase 3 with additional 14 key informants of the remote cancer centres. Purposeful sampling will be used for selection.

In phases 2 and 3, 105 healthcare providers will be recruited for the questionnaire survey in the CCCs. It will be assumed that each CCC (*n* = 7) has at least 15 healthcare providers involved into the care structures. In phase 3, healthcare providers in remote cancer centres will also be included in the survey. Based on the assumption that each CCC will reach additional seven remote cancer centres in phase 3 with five healthcare providers available for survey, a further 245 healthcare providers will be surveyed (total in phase 3: *n* = 350).

Purposeful sampling is used to conduct in-depth interviews with at least 18 healthcare providers in all phases.

For the qualitative interviews of patients, at least five patients at all CCCs and at least ten patients at remote cancer centres are recruited by purposeful sampling until saturation is reached. Sampling is informed by sex, age, tumour entity, exercise therapy, and experiences with the MOVE-ONKO care pathway as reported in patient questionnaires which are part of the evaluation of intervention outcomes.

#### Data analysis

Quantitative data are analysed using IBM SPSS 27 (or newer). Questionnaire scales are summarised according to the respective test manual guidelines. Variables are described as frequencies (categorical variables) or mean with standard deviation (metric variables). Differences between observations in the primary outcome (physical functioning) are analysed using a linear model with the value of physical functioning at t2 as dependent variable and its value at t0 as independent variable and adjusting for the cluster structure of the data (different centres) and patient characteristics (age, gender, education). Missing values will be replaced by multiple imputation. An additional sensitivity analysis will only use complete cases. Estimated parameters, *p* values and confidence intervals are reported. *P* values smaller than 0.05 will be considered statistically significant.

Other quantitative outcomes are analysed using the same analysis scheme as for the primary outcome.

The audio recordings of all individual interviews will be transcribed and pseudonymised. The transcripts will be analysed using the MAXQDA Analytics Pro software, following the structuring qualitative content analysis method by Kuckartz [41]. Two researchers will independently code and finally analysed by consensus. Main categories will be developed based on the interview guideline, and inductive subcategories will be developed during the coding process.

The results of the quantitative and qualitative surveys are finally brought together in evaluation workshops (triangulation).

#### Patient and public involvement

Patient involvement is a central part in the MOVE-ONKO studies. Besides a scientific and healthcare/insurance company counselling board, a counselling patient board meets every six months during the project time discussing relevant project development steps. Furthermore, representatives of all boards are involved in the curricula development of EOGs and EOSs and APP development.

## Discussion

If the implementation and evaluation of the MOVE-ONKO care pathway is successful, the aim is to transfer the pathway into standard care. In doing so, the MOVE-ONKO care pathway will address key barriers to the implementation of exercise therapy [42, 43].

Firstly, oncology patients will be comprehensively informed about the benefits and opportunities of exercise therapy and physical activity [1]. After comprehensive exercise counselling, they will be referred to needs-based exercise therapy programmes close to their home or they will be able to participate in online exercise therapy programmes.

Research has shown that exercise therapy advice combined with behavioural change techniques, delivered by healthcare providers, can improve patients' physical activity behaviour [44]. Furthermore, supervised exercise therapy shows greater effectiveness in terms of cancer- and cancer-related side effects than non-supervised programmes (e.g. home-based) [45]. However, MOVE-ONKO considers each patient's individual interest and personal barriers to exercise therapy, therefore home-based and online exercise therapy programmes are also available.

Secondly, the educational training of EOGs and EOSs will provide the necessary knowledge and technical support for healthcare providers to regularly advise exercise therapy to their cancer patients. In addition, comprehensive risk stratification allows for the referral of complex patients (e.g., those with severe symptoms or co-morbidities) to EOSs, reducing the complexity of the counselling service for EOGs [46].

Thirdly, we expect the development of regional exercise-based networks and multidisciplinary teamwork in the CCCs and remote cancer centres [47]. It will be of great interest to know which patients will be consulted by EOSs and which patients are transferred from EOGs to EOSs (e.g. due to high complexity or other issues). We expect different barriers and facilitators for CCCs due to different regional conditions and characteristics [18]. Overall, we predict an increase of the utilization of exercise therapy by patients through the involvement of multiprofessional healthcare providers in the MOVE-ONKO care pathway.

As there is currently no structured care pathway for exercise therapy in paediatric oncology, it is further planned to extend and adapt the MOVE-ONKO care pathway to paediatric cancer patients.

As independent cohorts of healthcare providers will be surveyed and interviewed, no within-person comparisons can be made. Key informant recruitment of healthcare providers may lead to social desirability bias. Outreach in phase 3 is hypothetical and it remains uncertain whether each CCC will reach an additional 7 remote cancer centres.

In the final phase of the study, the results will be published as a report. The results will be made available to the scientific community in the form of scientific publications and conference papers. Aggregated results of each CCCs implementation success will be made available to participating centres on request.

## Data Availability

No datasets were generated or analysed during the current study.

## Abbreviations

CCC: Comprehensive Cancer Centre
EOG: Exercise Oncology Guide
EOS: Exercise Oncology Specialist
HCPs: Healthcare Providers
KI: Key Informant
MOVE-ONKO: Multi-professional care pathway and network for the promotion of needs-oriented, resident-oriented exercise therapy for oncological patients (Multiprofessionelle Versorgungsstruktur und Netzwerk zur Förderung bedarfsorientierter, wohnortnaher Bewegungstherapie von onkologischen Patient*innen)
